# Comparative Analysis of Circulating Biomarkers for Patients Undergoing Resection of Colorectal Liver Metastases

**DOI:** 10.3390/diagnostics11111999

**Published:** 2021-10-27

**Authors:** Sven H. Loosen, Christoph Roderburg, Patrick H. Alizai, Anjali A. Roeth, Sophia M. Schmitz, Mihael Vucur, Mark Luedde, David Schöler, Pia Paffenholz, Frank Tacke, Christian Trautwein, Tom Luedde, Ulf P. Neumann, Tom F. Ulmer

**Affiliations:** 1 Clinic for Gastroenterology, Hepatology and Infectious Diseases, University Hospital Düsseldorf, Medical Faculty of Heinrich Heine University Düsseldorf, 40225 Düsseldorf, Germany; Christoph.Roderburg@med.uni-duesseldorf.de (C.R.); Mihael.Vucur@med.uni-duesseldorf.de (M.V.); david.schoeler@hhu.de (D.S.); Tom.Luedde@med.uni-duesseldorf.de (T.L.); 2 Department of Visceral and Transplantation Surgery, University Hospital RWTH Aachen, Pauwelsstrasse 30, 52074 Aachen, Germany; palizai@ukaachen.de (P.H.A.); aroeth@ukaachen.de (A.A.R.); sopschmitz@ukaachen.de (S.M.S.); uneumann@ukaachen.de (U.P.N.); fulmer@ukaachen.de (T.F.U.); 3 KGP Bremerhaven, Postbrookstraße 105, 27574 Bremerhaven, Germany; mark.luedde@web.de; 4 Department of Urology, University Hospital Cologne, Kerpener Str. 62, 50937 Cologne, Germany; pia.paffenholz@uk-koeln.de; 5 Department of Hepatology and Gastroenterology, Charité University Medicine Berlin, Augustenburger Platz 1, 13353 Berlin, Germany; frank.tacke@charite.de; 6 Department of Medicine III, University Hospital RWTH Aachen, Pauwelsstrasse 30, 52074 Aachen, Germany; ctrautwein@ukaachen.de; 7 Department of Surgery, Maastricht University Medical Centre (MUMC), 5800 Maastricht, The Netherlands

**Keywords:** CRLM, CRC, cancer, liver resection, CEA, CA19-9, CRP, survival

## Abstract

Surgical tumor resection has evolved as a potentially curative therapy for patients with resectable colorectal liver metastases (CRLM). However, disease recurrence is common and the available preoperative stratification strategies are often imprecise to identify the ideal candidates for surgical treatment, resulting in a postoperative 5-year survival rate below 50%. Data on the prognostic value of CEA, CA19-9 and other common laboratory parameters after CRLM resection are scarce and partly inconclusive. Here, we analyzed the prognostic potential of circulating CEA and CA19-9 in comparison to other standard laboratory markers in resectable CRLM patients. Serum levels of tumor markers and other laboratory parameters were analyzed in 125 patients with CRLM undergoing tumor resection at a tertiary referral center. Results were correlated with clinical data and outcome. Both tumor markers were significantly elevated in CRLM patients compared to healthy controls. Interestingly, elevated levels of CEA, CA19-9 and C-reactive protein (CRP) were associated with an unfavorable prognosis after CRLM resection in Kaplan–Meier curve analysis. However, only CEA and not CA19-9 or CRP serum levels were an independent prognostic marker in multivariate Cox regression analysis. Our data demonstrate that circulating levels of CEA rather than CA19-9 might be a valuable addition to the existing preoperative stratification algorithms to identify patients with a poor prognosis after CRLM resection.

## 1. Introduction

Colorectal cancer (CRC) represents one of the most common types of cancer worldwide. In 2012, there were 447,000 new cases of CRC in Europe and over 1.4 million new cases worldwide, leading to 694,000 deaths [1]. Despite the major efforts of cancer prevention and early cancer diagnosis, up to 25% of patients present with liver metastases at the time of diagnosis, and another 25% of patients develop metastases during the clinical course [2]. Over the last decade, the clinical outcome of patients with metastatic CRC (mCRC) has significantly improved [1,3]. In clinical phase III trials, a median overall survival of up to 45 months could be achieved [3]. Beside other improvements in the management of mCRC patients, the improved outcome mainly results from a “continuum of care” for these patients, incorporating highly effective systemic and local ablative therapies as well as aggressive tumor resection strategies, offering the chance of cure or at least durable, relapse-free survival [4]. Nevertheless, even after successful, curatively indented resection of colorectal liver metastases (CRLM), about 65% of patients develop hepatic relapse within three years after surgery [2,5,6]. In this context, decisions for or against tumor resection in CRLM patients are often conflictive and challenging. Current guidelines recommend that both “oncological“(prognostic) and “technical“ (surgical) criteria should be considered when evaluating patients for surgery [4,7,8]. However, both terms are only poorly defined, and prospective evaluations are missing, leaving the physician with a high degree of uncertainty when evaluating whether a CRLM patient will actually benefit from surgical resection in terms of overall survival. 

As fully disease-specific biomarkers for CRC have not been established, carcinoembryonic antigen (CEA) represents the standard tumor marker in patients with colorectal cancer [9]. CEA, which is one parameter of the well-established Fong score, is the most frequently used biomarker for treatment predictive purposes in CRC patients undergoing liver resection or receiving chemotherapy [9,10]. Besides CEA, CA19-9 has also been suggested as a prognostic marker for CRC patients [9]. Nevertheless, serum levels of CA19-9 are also elevated in patients with a broad variety of gastrointestinal tumor diseases and with non-malignant biliary diseases, such as primary sclerosing cholangitis or biliary obstruction due to choledocholithiasis [11,12], implying that CA19-9 might not be the ideal marker in patients with CRC. 

In the present study, we therefore evaluated the potential role of CA19-9, CEA, CRP and other routinely measured laboratory parameters in a large cohort of 125 CRLM patients, who underwent surgical tumor resection with curative intent at our university hospital between 2011 and 2017. 

## 2. Materials and Methods

### 2.1. Study Design and Patient Characteristics

In this observational cohort study, we evaluated the role of CA19-9, CEA, CRP and other laboratory parameters as diagnostic and prognostic serum markers in patients undergoing resection of CRLM. A total of 125 patients who were admitted for CRLM resection at University Hospital RWTH Aachen between 2011 and 2017 were included in this study. As a control group, 50 healthy blood donors who were medically examined on a regular basis and showed no sign of hepatic disease were included. The study protocol was approved by the local ethics committee and conducted in accordance with the ethical standards laid down in the Declaration of Helsinki (EK 206/09, approval date: 5 January 2010, ethics committee of the University Hospital RWTH Aachen, RWTH University, Aachen, Germany). Written informed consent was obtained from the patients. 

### 2.2. Measurement of Laboratory Parameters

All laboratory markers were analyzed in the central laboratory at University Hospital RWTH Aachen. Circulating levels of CEA and CA19-9 were analyzed with an electrochemiluminescence immunoassay (ECLIA) using the Cobas 8000 e602 modular analyzer series (Hoffmann-La Roche AG, Basel, Switzerland). Standard hematological and clinical chemistry parameters were measured using the Sysmex XN9000 (Sysmex GmbH, Norderstedt, Germany) and the Cobas 8000 c701 (Hoffmann-La Roche AG, Basel, Switzerland).

### 2.3. Statistical Analysis

Serum data are given as median and range. Kolmogorov–Smirnov and Shapiro–Wilk tests were used to test for normal distribution. Non-parametric data were compared using the Mann–Whitney U test and, for multiple comparisons, the Kruskal–Wallis test. Box plot graphics display a statistical summary of the median, quartiles and ranges. Correlation analyses were performed using the Spearman correlation tests. ROC curves were generated by plotting sensitivity against 1-specificity. The optimal cut-off values for ROC curves were established using the Youden index (YI = sensitivity + specificity − 1). Kaplan–Meier curves were plotted to display the impact on survival. A log-rank test was used to test for differences between subgroups in Kaplan–Meier curve analysis. The prognostic value of the variables was further tested by univariate and multivariate analysis in the Cox regression model. The inclusion criterion for multivariate testing was a *p*-value < 0.25 in univariate analysis. All statistical analyses were performed with SPSS 23 (SPSS, Chicago, IL, USA) [13]. A *p*-value of < 0.05 was considered statistically significant (* *p* < 0.05; ** *p* < 0.01; *** *p* < 0.001).

## 3. Results

### 3.1. Patient Characteristics

A total of 125 patients who underwent surgical resection of CRLM at University Hospital RWTH Aachen were included into this study. The median age was 63 years (range: 25–85 years). A total of 64.8% of patients were male and 35.2% were female. In 18.5% of patients, CRLM originated from right-sided CRC, while 81.5% initially presented with left-sided CRC. During the follow-up period, 41.6% of patients became deceased. The median overall survival of our study cohort was 1318 days. Patient characteristics are summarized in Table 1.

### 3.2. Serum Levels of CEA and CA19-9 Are Elevated in Patients with CRLM

**Figure 1:**
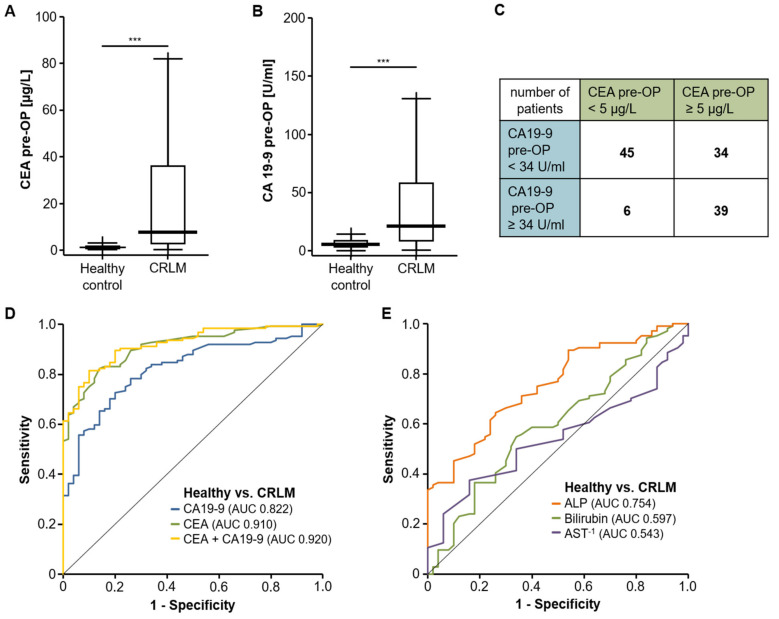
Serum levels of CEA and CA19-9 are elevated in patients with CRLM. Preoperative serum levels of CEA (**A**) and CA19-9 (**B**) are significantly elevated in patients with CRLM compared to healthy controls. (**C**) Number of patients with elevated CEA and CA19-9 levels above the standard cut-off value. (**D**) ROC curve analysis reveals AUC values of 0.910 and 0.822 for CEA and CA19-9, respectively, for the differentiation between CRLM patients and healthy controls. (**E**) Other routinely tested serum markers of liver injury have an inferior AUC. *** *p* < 0.001.

We first compared pre-operative serum levels of CEA and CA19-9 between patients with CRLM (*n* = 124) and healthy controls (*n* = 50). Both tumor markers were significantly higher in CRLM patients (Figure 1A,B, Table 2). In contrast, the median serum level of CRP and the leucocyte count were within the range of normal (Table 2). When we applied our laboratory’s standard cut-off values (CEA: 5 µg/L, CA19-9: 34 U/mL), of the 124 patients with available tumor marker levels, 34 patients (27.4%) displayed an isolated elevation of CEA and 6 patients (4.8%) had exclusively elevated levels of CA19-9. In total, 39 CRLM patients (31.5%) showed an elevation above the standard cut-off values for both tumor markers, while 45 patients (36.3%) presented with normal pre-operative CEA and CA19-9 values (Figure 1C). Subsequently, we tested the diagnostic value of both markers in ROC curve analysis, which showed an AUC of 0.910 and 0.822 for CEA and CA19-9, respectively, regarding the differentiation between CRLM patients and healthy controls (Figure 1D). At the ideal cut-off value of 2.55 µg/L, CEA serum levels had a diagnostic sensitivity of 82.3% with specificity of 86.0%, while the ideal CA19-9 cut-off value (8.95 U/mL) showed a sensitivity and specificity of 72.6% and 80.0%, respectively. Notably, the combination of CEA and CA19-9 revealed an even higher diagnostic accuracy with an AUC of 0.920 (Figure 1D). In contrast, standard liver laboratory parameters such as bilirubin, AST or ALP had an inferior power to discriminate between CRLM patients and healthy controls, showing AUC values of 0.754 (ALP), 0.597 (bilirubin) and 0.543 (AST^−1^) (Figure 1E). 

### 3.3. CEA, CA19-9 and CRP Serum Levels Correlate with Tumor Size of CRLM

**Figure 2:**
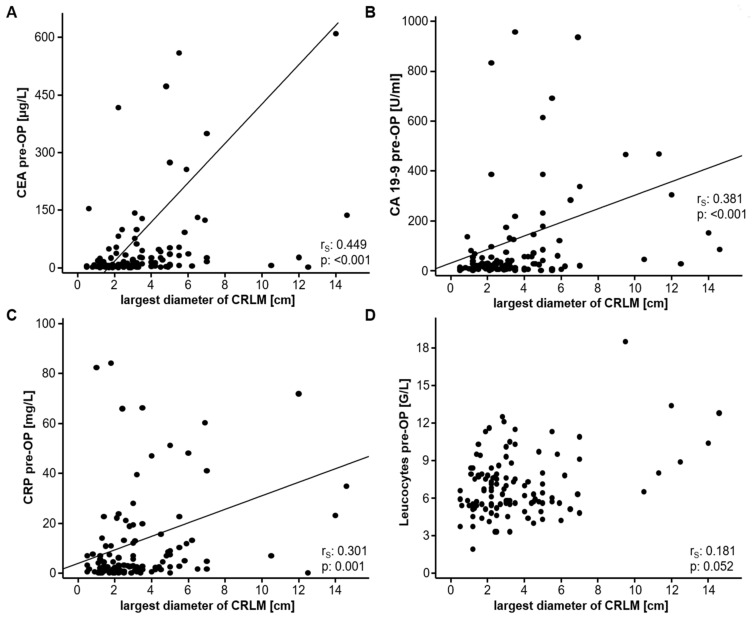
CEA, CA19-9 and CRP serum levels correlate with the size of CRLM. Serum levels of CEA (**A**), CA19-9 (**B**) and CRP (**C**) show a strong correlation with the size of CRLM. The leucocyte count does not significantly correlate with the CRLM size (**D**).

To unravel a potential correlation between circulating levels of tumor markers (CEA and CA19-9) as well as inflammatory parameters (CRP and leucocytes), which have also been suggested to have prognostic relevance in CRC patients, and the size of CRLM in our cohort of patients, we subsequently assessed the largest diameter of the CRLM in the resected liver samples. Interestingly, serum levels of CEA and CA19-9 showed a strong positive correlation with the tumor size of CRLM (Figure 2A,B). In line with this, CRP serum levels also correlated with the largest diameter of CRLM (Figure 2C), while statistical significance was not fully reached with respect to the leucocyte count (Figure 2D). 

We next evaluated if serum levels of CEA, CA19-9, CRP or leucocyte count differed in patients with distinct CRC disease characteristics. Based on recent data on a pivotal role of CRC tumor localization [14], we first compared patients whose CRLM originated from right-sided CRC and patients with left-sided primary disease. However, we did not find a significant difference in serum CEA (Figure S1A), CA19-9 (Figure S1B) and CRP (Figure S1C) levels or the leucocyte count (Figure S1D) between these subgroups of patients. Similarly, *KRAS* mutated patients showed unaltered levels of CEA, CA19-9, CRP and leucocytes when compared to *KRAS* wild-type CRC patients (Figure S1E–H). Finally, we assessed if the ECOG performance status (PS) might be reflected by circulating biomarker levels. However, we observed no differences in CEA, CA19-9, CRP or leucocyte levels in patients with normal (ECOG 0) or impaired (ECOG 1/2) PS (Figure S2A–D).

### 3.4. CEA Is an Independent Predictor of Long-Term Survival after CRLM Resection

**Figure 3:**
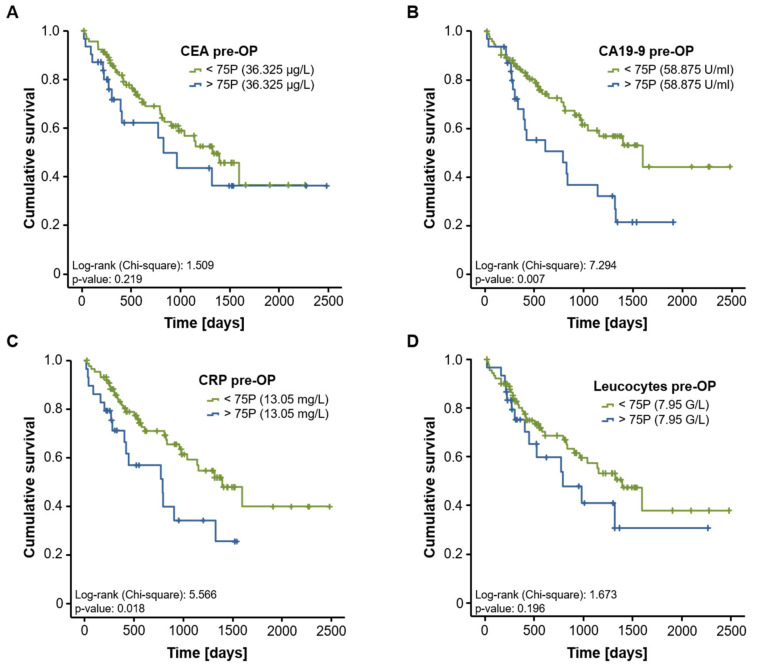
Evaluation of CEA, CA19-9, CRP and leucocyte count as prognostic marker after CRLM resection. Kaplan–Meier curve analysis reveals that only CA19-9 (**B**) and CRP (**C**), but not CEA (**A**) or leucocyte count (**D**), indicate an impaired long-term survival in patients with circulating levels above the 75th percentile.

**Figure 4:**
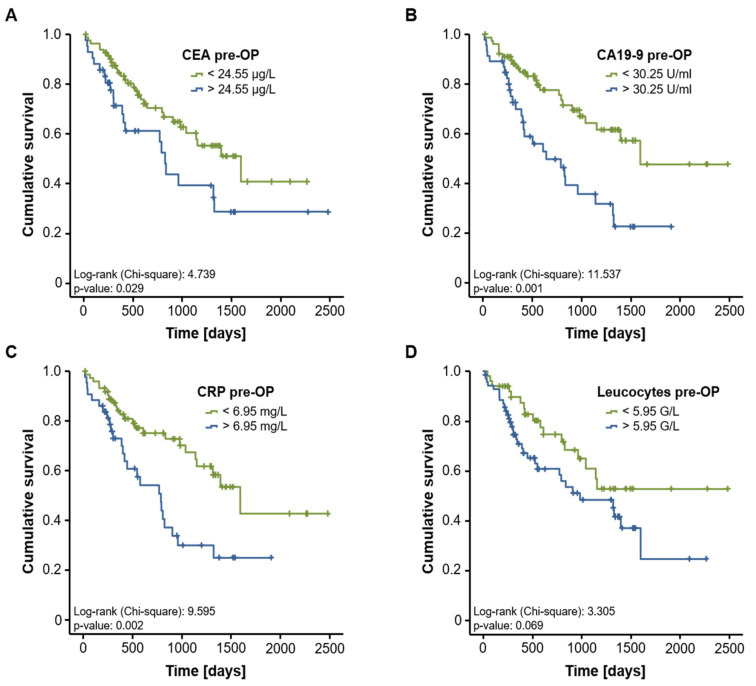
CEA, CA19-9 and CRP are prognostic factors of overall survival after resection of CRLM. When using the optimal prognostic cut-off value, Kaplan–Meier curve analyses show a significant impaired long-term survival for CRLM patients with CEA serum levels above 24.55 µg/L (**A**), CA19-9 serum levels above 30.25 U/mL (**B**) and CRP levels above 6.95 mg/L (**C**). The ideal cut-off value for the leucocyte count (5.95 G/L) is unable to discriminate between long-term survivors and non-survivors (**D**).

Based on these results, we subsequently analyzed a potential prognostic role of CEA, CA19-9, CRP and leucocyte count in our study population. We therefore divided our cohort of patients into two subgroups according to the pre-operative levels of the respective biomarker (above or below the 75th percentile). When using these cut-off values, Kaplan–Meier curve analysis revealed that high CA19-9 but not CEA serum levels identified patients with a significantly impaired prognosis following CRLM resection (Figure 3A,B). Similarly, patients with high pre-operative CRP serum levels (above the 75th percentile) had an unfavorable postoperative survival (Figure 3C), while the leucocyte count was unable to discriminate between survivors and non-survivors (Figure 3D). Subsequently, we established ideal prognostic cut-off values for each biomarker using the Youden index method. Importantly, CEA serum levels at the optimal cut-off value of 24.55 µg/L now significantly discriminated between patients with a good postoperative prognosis and patients that succumbed to death early (Figure 4A). The ideal CA19-9 (30.25 U/mL) and CRP (6.95 mg/L) cut-off values resulted in further increased prognostic power of these serum markers (Figure 4B,C), while statistical significance was not reached for the leucocyte count at the ideal cut-off value of 5.95 G/L (Figure 4D). 

To further unravel the prognostic potential of the analyzed biomarkers, we finally performed Cox regression analysis. In univariate analyses, including tumor markers (CEA and CA19-9), markers of inflammation (CRP and leucocyte count), standard parameters of liver (bilirubin, AST, ALT, ALP) and kidney (creatinine) function, tumor characteristics (tumor size, CRC localization, *KRAS* status) and distinct clinical parameters (age, BMI, ECOG PS), only CEA, CA19-9 and CRP, as well as AST and ALT, were prognostic factors after CRLM resection (Table 3). Importantly, in multivariate analysis, only serum levels of CEA, but not CA19-9 or CRP, stood out as an independent prognostic factor for long-term survival in this setting (Table 3).

## 4. Discussion

Carcinoembryonic antigen (CEA) represents a glycoprotein with a proven function in cell adhesion mechanisms during the fetal development of the gastrointestinal tract [15]. Since, physiologically, CEA is not produced after birth, elevated CEA serum concentrations are in almost all cases indicative of the presence of a malignant disease [16,17]. As such, CEA is routinely used as a tumor marker in the diagnostic workup and surveillance of patients with colorectal carcinoma [9]. Here, we demonstrate that serum levels of CEA are significantly elevated in patients with CRLM before curative intended tumor resection and indicate the presence of a CRLM with a higher sensitivity and specificity than other frequently used markers for CRC. 

CEA is the most widely used tumor marker in patients with colorectal cancer. Compared to other potential predictive markers, measurement of serum CEA levels is inexpensive, standardized, widely used and easily performed [18]. In recent years, many studies have focused on the predictive value of CEA levels in patients with colorectal cancer receiving chemotherapy or chemoradiotherapy [19,20,21]. Most studies showed that low pre-treatment CEA levels are associated with a good patients’ outcome [21], but only very few data are available on the predictive role of CEA measurements in CRLM patients undergoing curatively intended liver surgery, supporting further analyses addressing this question in large and well-characterized cohorts of patients [22,23]. In contrast to many other metastasized tumors, long-term survival can be attained in up to 50% of patients with colorectal liver metastases (CRLM) when complete metastasectomy is performed [2,5]. Despite only few randomized data comparing surgical and non-surgical disease management being available, surgery has risen to become the “Golden Standard” in patients with CRLM. Current guidelines recommend that both “oncological” (prognostic) and “technical” (surgical) criteria should be considered when evaluating the patients for surgery [4,7,8]. The “technical” definitions of resectable CRLM mainly depends on the future liver remnant or a remnant-liver-to-body weight ratio of >0.5 after complete tumor resection [24]. However, even if complete tumor resection is performed, about half of the patients will develop recurrent systemic disease within 3 years of resection, suggesting that not all CRLM patients will benefit from extensive liver surgery and that appropriate patient selection remains the key factor in the surgical management of CRLM patients [25]. In this context, different preoperative assessment algorithms (including imaging, liver function tests and clinical performance status) have been proposed; however, since most of these algorithms face the lack of an appropriate prospective validation, it has remained challenging to predict which individual patients will actually benefit from extended liver surgery in terms of postoperative overall survival (OS) [26]. Thus, oncological criteria reflecting the tumor biology might provide important information for clinical decision making and might be a valuable addition to the existing stratification algorithms for patients with CRLM [26]. Here, we show in a large cohort of CRLM patients undergoing tumor resection at a tertiary referral center that preoperative levels of circulating CEA represent an independent predictor of patients’ survival and discriminate between long-term survivors and non-survivors in Kaplan–Meier curve analysis. In line with our results, it was recently demonstrated in another cohort of CRLM patients that elevated concentrations of CEA after liver resection were also associated with shorter OS [18]. Similarly, elevated levels of CA19-9, another biomarker frequently used in the context of gastrointestinal malignancies, had an indicative role for an impaired patients’ outcome [18]. In our analysis, elevated CA19-9 levels were indicative for a poor outcome according to Kaplan–Meier curve analysis; however, statistical significance for the prediction of long-term survival was not reached in multivariate Cox regression analysis (see Table 3). Along with CEA and CA19-9, systemic markers of inflammation were recently suggested as a prognostic factor in patients undergoing tumor resection for CRLM [27,28]. We show that preoperative CRP serum levels are a predictive factor for long-term survival in Kaplan–Meier curve analysis and univariate (but not multivariate) Cox regression analysis. Interestingly, in contrast to CRP, a pathological leukocyte count was not associated with an impaired patients’ survival in our cohort of patients. 

Just recently, the primary CRC tumor localization was identified as a both prognostic and predictive marker in the treatment of patients with metastasized CRC [29,30,31]. CRLM patients with a right-sided tumor display a significantly impaired outcome following tumor resection [29,30,31]. In our analysis, we demonstrate that the serum levels of CEA were similar in patients with right- or left-sided CRC. Consequently, the prognostic value of this marker was independent of the primary tumor location in multivariate Cox regression analysis (see Table 3). Similarly, concentrations of CEA were independent of the mutational status of the patients since *KRAS* mutated patients displayed almost identical levels of CEA as wild-type patients. Finally, we analyzed whether differences in overall patients’ performance state might have biased our analysis. However, since patients with a better or worse performance state displayed similar levels of CEA, it seems unlikely that such a bias is present. In summary, our data indicate that CEA represents a robust biomarker reflecting the patients’ prognosis independent of other tumor-specific factors and might thus be used in all CRLM patients to estimate the outcome after surgery. 

We acknowledge some limitations of our study. Importantly, this study only considers the prognosis of CRLM patients after extended liver surgery. Of note, it was not analyzed whether alternative treatment options would have led to a more (or less) favorable outcome. Thus, it is not possible to draw any conclusions on the question of whether patients with elevated CEA levels (and an unfavorable prognosis) might have benefited to a greater extent from locally ablative techniques, systemic therapy or even a best supportive care approach. In addition, detailed information on perioperative chemotherapy was not available, and we therefore cannot draw any conclusion about its effect on overall survival. Finally, subgroup analysis of rectal vs. colon cancer patients was not feasible due to the relatively small cohort size. Especially in terms of the emerging evidence of a distinct underlying tumor biology of the different CRC localizations, further analyses are warranted to fully dissect the role of the analyzed tumor markers in these individual patient populations. Nevertheless, our present study provides evidence that CEA serum levels should be considered as a prognostic marker in patients undergoing CRLM resection and might therefore be a valuable addition to the existing preoperative stratification algorithms in the future. Similar results have recently been published by the group of Margonis et al. [4,32], further highlighting the validity of the data presented here and arguing for a clinical use of CEA in the prognosis estimation of patients with CRLM. 

## Figures and Tables

**Table 1:** Characteristics of study population.

	Study Population
CRLM patients	125
Sex [%]: male–female	64.8–35.2
Age [years, median and range]	63 (25–85)
BMI [kg/m^2^, median and range]	25.5 (17.40–38.74)
Tumor characteristics	
Largest diameter of CRLM [cm, median and range]:	2.85 (0.5–14.6)
CRC localization [%, right- vs. left-sided]	18.5–81.5
KRAS status [%, wild-type vs. mutated]	57.4–42.6
ECOG PS [%]	
0	66.4
1	32.0
2	1.6
Death during follow up [%]:	
yes–no	41.6–58.4

**Table 2:** Serum levels of laboratory markers.

	CRLM Patients Median [Range]	Healthy Controls Median [Range]
CEA [µg/L]	7.65 [0.3–2703.0]	1.25 [0.3–6.3]
CA19-9 [U/mL]	20.9 [0.6–4708.0]	5.4 [0–44.1]
Leucocytes [G/L]	6.6 [1.9–18.5]	-
CRP [mg/L]	3.2 [0–120.6]	-
AST [U/L]	28.0 [2.1–399.0]	28.0 [20.0–78.0]
ALT [U/L]	23.5 [8.0–180.0]	-
GGT [U/L]	53.0 [10.0–1708.0]	-
ALP [U/L]	87.5 [41.0–479.0]	65.0 [36.0–102.0]
Bilirubin [mg/dL]	0.5 [0.1–1.29]	0.41 [0.1–1.46]
Creatinine [mg/dL]	0.84 [0.46–1.4]	-
Sodium [mmol/L]	140.0 [128.0–147.0]	-
Potassium [mmol/L]	4.4 [2.6–5.9]	-
Calcium [mmol/L]	2.33 [1.26–3.15]	-
Haemoglobin [g/L]	13.2 [8.2–16.9]	-
Platelets [cells/nl]	236.0 [102.0–782.0]	-

CEA: carcinoembryonic antigen, CA 19-9: carbohydrate-Antigen 19-9, CRP: C-reactive protein, AST: aspartate transaminase, ALT: alanine transaminase, GGT: γ-Glutamyl transpeptidase, ALP: alkaline phosphatase.

**Table 3:** Univariate and multivariate Cox regression analyses for the prediction of long-term survival.

	Univariate Cox Regression		Multivariate Cox Regression	
Parameter	*p*-Value	Hazard-Ratio [95% CI]	*p*-Value	Hazard-Ratio [95% CI]
CEA	<0.001	1.001 [1.001–1.002]	0.001	1.002 [1.001–1.003]
CA19-9	0.001	1.001 [1.000–1.001]	0.166	1.000 [1.000–1.001]
CRP	0.002	1.016 [1.006–1.027]	0.799	0.998 [0.980–1.016]
Leucocytes	0.055	1.121 [0.998–1.259]	0.133	1.129 [0.964–1.321]
Creatinine	0.667	0.730 [0.174–3.066]		
Bilirubin	0.222	0.448 [0.123–1.628]	0.163	0.321 [0.065–1.585]
AST	0.017	1.005 [1.001–1.009]	0.063	1.006 [1.000–1.013]
ALT	0.250	1.007 [0.995–1.019]		
ALP	0.010	1.004 [1.001–1.006]	0.264	1.002 [0.998–1.006]
Tumor size (largest diameter of CRLM)	0.150	1.071 [0.976–1.176]	0.580	0.996 [0.854–1.092]
CRC Localization (right- vs. left-sided)	0.124	0.608 [0.322–1.146]	0.167	0.581 [0.270–1.254]
KRAS status (*KRAS* wild-type vs. *KRAS* mutation)	0.397	0.771 [0.322–1.568]		
Age	0.262	1.016 [0.998–1.044]		
BMI	0.451	1.023 [0.965–1.084]		
ECOG PS (ECOG 0 vs. ≥1)	0.464	1.234 [0.702–2.169]		

CEA: carcinoembryonic antigen, CA 19-9: carbohydrate-Antigen 19-9, CRP: C-reactive protein, AST: aspartate transaminase, ALT: alanine transaminase, ALP: alkaline phosphatase, CRLM: colorectal liver metastases, CRC: colorectal cancer, BMI: body mass index, ECOG PS: ECOG performance status.

## Data Availability

Data are available upon reasonable request from the corresponding author.

## Supplementary Materials

The following are available online at https://www.mdpi.com/article/10.3390/diagnostics11111999/s1, Figure S1: Evaluation of biomarker levels and tumor characteristics. Circulating levels of CEA (A), CA19-9 (B) and CRP (C) as well as the leucocyte count (D) are unaltered between patients with initial right- or left-sided CRC. Patients with *KRAS* mutation have similar levels of CEA (E), CA19-9 (F) and CRP (G) as well as leucocyte count (H) compared to *KRAS* wild-type patients. Figure S2: Evaluation of biomarker levels and the ECOG performance status. Circulating levels of CEA (A), CA19-9 (B) and CRP (C) as well as the leucocyte count (D) are unaltered between patients with normal (ECOG O) or impaired (ECOG 1/2) performance status.
