# Supplementary figures and images for: Comparative Analysis of Circulating Biomarkers for Patients Undergoing Resection of Colorectal Liver Metastases
Source: Diagnostics (Basel). 2021 Oct 27;11(11):1999. doi: 10.3390/diagnostics11111999 (PMC8622404; doi:10.3390/diagnostics11111999)

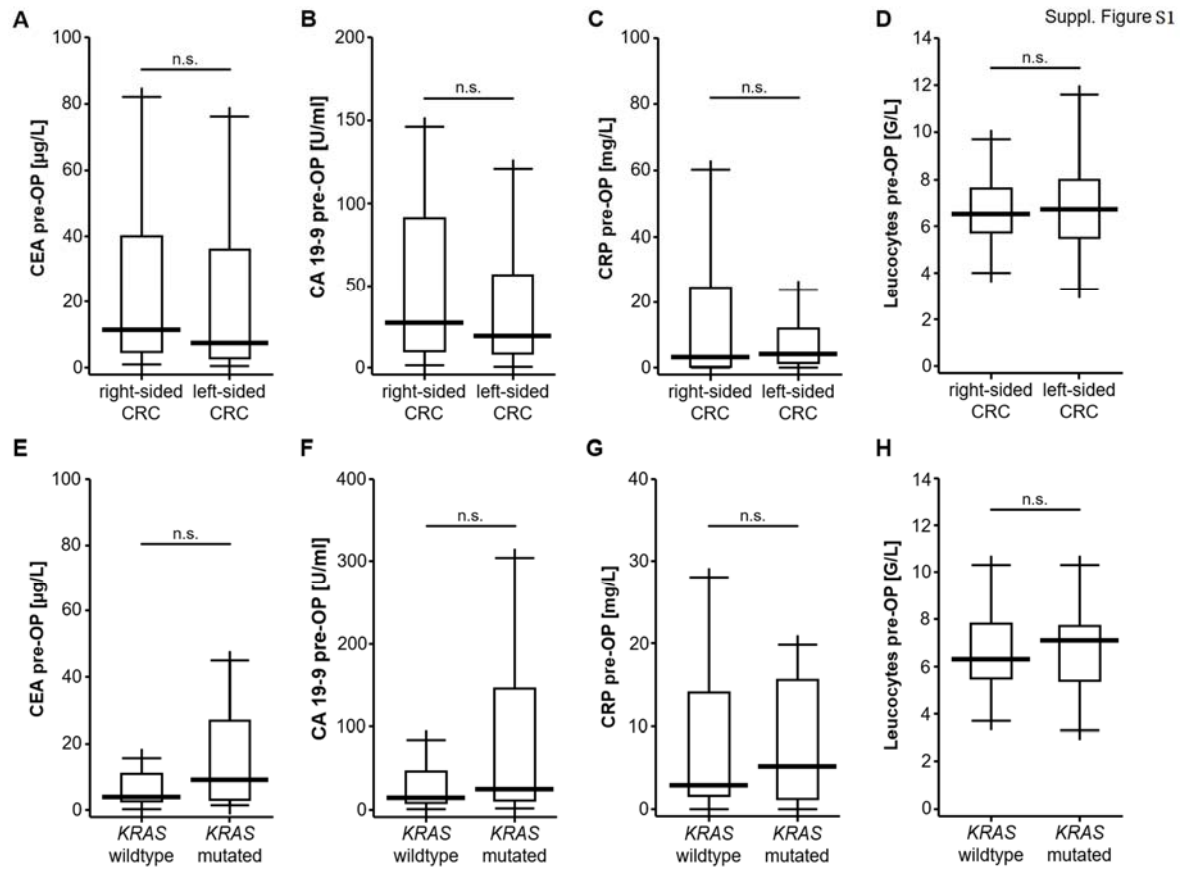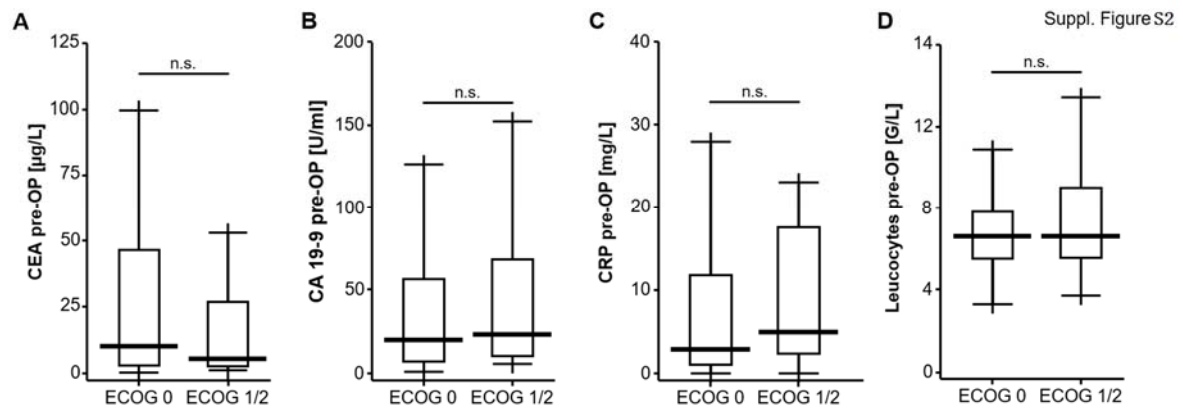

Supplement: Supplementary file 1 [file diagnostics-11-01999-s001.zip › diagnostics-1390365-supplementary.pdf]
